# Nasopharyngeal Meningococcal Carriage among Children and Adolescents in Turkey in 2018: An Unexpected High Serogroup X Carriage

**DOI:** 10.3390/children8100871

**Published:** 2021-09-29

**Authors:** Mahmut Can Kizil, Omer Kilic, Mehmet Ceyhan, Merve Iseri Nepesov, Adem Karbuz, Zafer Kurugol, Mustafa Hacimustafaoglu, Solmaz Celebi, Meltem Dinleyici, Kursat Bora Carman, Cihangul Bayhan, Yasemin Balliel, Murat Sutcu, Necdet Kuyucu, Meda Kondolot, Soner Sertan Kara, Sevliya Ocal Demir, Ummuhan Cay, Zeynep Gokce Gayretli Aydin, Mucahit Kaya, Ener Cagri Dinleyici

**Affiliations:** 1 Division of Pediatric Infectious Diseases, Faculty of Medicine, Eskisehir Osmangazi University, Eskisehir 26040, Turkey; mcankizil@hotmail.com (M.C.K.); omerkilic7@yahoo.com (O.K.); iserimerve@yahoo.com (M.I.N.); 2 Division of Pediatric Infectious Diseases, Faculty of Medicine, Hacettepe University, Ankara 06230, Turkey; mceyhan@hacettepe.edu.tr; 3 Tascioglu City Hospital Division of Pediatric Infectious Diseases, Istanbul 34000, Turkey; karbuzadem@hotmail.com; 4 Division of Pediatric Infectious Diseases, Faculty of Medicine, Ege University, Izmir 35000, Turkey; zkurugol@gmail.com; 5 Division of Pediatric Infectious Diseases, Faculty of Medicine, Uludag University, Bursa 16059, Turkey; mkemal@uludag.edu.tr (M.H.); solmaz@uludag.edu.tr (S.C.); 6 Division of Social Pediatrics, Faculty of Medicine, Eskisehir Osmangazi University, Eskisehir 26040, Turkey; meltemayata@hotmail.com; 7 Division of Pediatric Neurology, Faculty of Medicine, Eskisehir Osmangazi University, Eskisehir 26040, Turkey; kbcarman@gmail.com; 8 Division of Pediatric Infectious Diseases, Gulhane Training and Research Hospital, Ankara 06300, Turkey; cihangulbayhan@gmail.com; 9 Antalya Muratpaşa Çaybaşı No:1 Family Health Center, Antalya 07000, Turkey; yaseminballiel@gmail.com; 10 Division of Pediatric Infectious Diseases, Faculty of Medicine, Istinye University, Istanbul 34010, Turkey; sutcu13@yahoo.com; 11 Division of Pediatric Infectious Diseases, Faculty of Medicine, Mersin University, Mersin 33343, Turkey; nkuyucu@yahoo.com; 12 Division of Social Pediatrics, Faculty of Medicine, Erciyes University, Kayseri 38039, Turkey; medakondolot@gmail.com; 13 Division of Pediatric Infectious Diseases, Faculty of Medicine, Aydin Adnan Menderes University, Aydin 09010, Turkey; drsoner@yahoo.com; 14 Division of Pediatric Infectious Diseases, Faculty of Medicine, Istanbul Medeniyet University, Istanbul 34000, Turkey; sevliyademir@gmail.com; 15 Division of Pediatric Infectious Diseases, Faculty of Medicine, Cukurova University, Adana 01330, Turkey; ucay-1205@hotmail.com; 16 Division of Pediatric Infectious Diseases, Faculty of Medicine, Karadeniz Technical University, Trabzon 61080, Turkey; zggayretli@gmail.com; 17 Diagen Biotechnology, Ankara 06710, Turkey; m.kaya@diagen.com.tr; 18 Department of Pediatrics, Faculty of Medicine, Ener Cagri Dinleyici, Eskisehir Osmangazi University, Eskisehir 26040, Turkey

**Keywords:** *Neisseria meningitidis*, carriage, meningococcal carriage, serogrup X, MenX

## Abstract

Meningococcal carriage studies and transmission modeling can predict IMD epidemiology and used to define invasive meningococcal disease (IMD) control strategies. In this multicenter study, we aimed to evaluate the prevalence of nasopharyngeal *Neisseria meningitidis* (*Nm)* carriage, serogroup distribution, and related risk factors in Turkey. Nasopharyngeal samples were collected from a total of 1267 children and adolescents and were tested with rt-PCR. *Nm* carriage was detected in 96 participants (7.5%, 95% CI 6.1–9.0), with the peak age at 13 years (12.5%). Regarding age groups, Nm carriage rate was 7% in the 0–5 age group, was 6.9%in the 6–10 age group, was 7.9% in the 11–14 age group, and was 9.3% in the 15–18 age group. There was no statistically significant difference between the groups (*p* > 0.05). The serogroup distribution was as follows: 25% MenX, 9.4% MenA, 9.4% MenB, 2.1% MenC, 3.1% MenW, 2.1% for MenY, and 48.9% for non-groupable. The *Nm* carriage rate was higher in children with previous upper respiratory tract infections and with a high number of household members, whereas it was lower in children with antibiotic use in the last month (*p* < 0.05 for all). In this study, MenX is the predominant carriage strain. The geographical distribution of Nm strains varies, but serogroup distribution in the same country might change in a matter of years. Adequate surveillance and/or a proper carriage study is paramount for accurate/dynamic serogroup distribution and the impact of the proposed vaccination.

## 1. Introduction

Invasive meningococcal disease (IMD) caused by *Neisseria meningitidis (Nm)* is rare, yet it represents a worldwide public health issue. Despite successful antibiotic and supportive therapies, about 10% of patients die, and survivors may experience catastrophic consequences, such as amputation, neurologic and behavioral problems, and hearing loss [1]. There are 12 known capsular serogroups, with six of them (A–C, W, X, and Y) causing the majority of human illness [1,2,3]. The incidence of IMD and the epidemiology of serogroups vary by age group, geographic region, and immunization strategies [1,4,5]. Appropriate monitoring is essential for accurate epidemiological data and, consequently, the implementation of effective prevention programs [1].

Approximately 10% of the population has *Nm* as a carrier in their nasopharynx at any given time, and IMDs are primarily spread through carriers. Carriage is typically asymptomatic, but the pathogen can occasionally invade the mucosa and enter the bloodstream, resulting in IMD, which occurs shortly after the bacteria are acquired [6]. *Nm* carriage is found in 3–20% of the population′s healthy people [3]. *Nm* carriage has been linked to age, crowded living conditions, cigarette smoke exposure, a history of respiratory infection, certain adolescent social behavior, and antibiotic use [4,6,7,8,9,10]. Adolescents and young adults have the highest carriage rate [7]. While meningococcal colonization is the first stage in the development of IMD, little is known about the risk variables that predict progression from asymptomatic to symptomatic conditions. The causal serogroup, bacterial genomic plasticity, the human immune system, and environmental variables all play a role in the development of IMD from carriage [3].

IMD and *Nm* carriage serogroup distribution varies by geographical region and changes over time within the same geographical region [11]. The seroepidemiology of meningococcal illness in Turkey is dynamic and distinct from that in other countries [12]. According to a surveillance study, *Nm* is the most common cause of bacterial meningitis in children, with MenB being the most common strain in Turkey between 2005 and 2018, followed by MenW, MenA, and MenY [13,14]. A meningococcal vaccine is not included in Turkey’s national childhood immunization program. Military personnel and Hajj/Umrah pilgrims have routinely received quadrivalent meningococcal conjugated vaccination (MenACWY). MenACWY and the four-component MenB vaccine (4CMenB) are accessible through private practice.

The frequency of carriers and serogroups by age group is critical for determining disease burden, predicting outbreaks, and developing an effective immunization strategy [1]. Immunization efforts tailored to a particular country or region usually target the populations most at risk of IMD or transmission. In 2015, we conducted a statewide multi-center study of nasopharyngeal meningococcal carriage among adolescents and young adults aged 10 to 25 years, and we found that the meningococcal carriage rate was 6.3% with MenW predominance [15]. We propose to examine the defined *Nm* circulation carriage rate among children and adolescents aged 0 to 18 years in the same cities two years following this investigation, as well as to identify the risk variables linked with carriage and compare meningococcal serogroup epidemiology with the 2015 study results.

## 2. Materials and Methods

### 2.1. Study Population and Sampling

Twelve city centers that represent the country in terms of population and geography were determined. Thirteen health care centers in these 12 cities (Istanbul, Ankara, Izmir, Bursa, Antalya, Konya, Mersin, Diyarbakir, Kayseri, Eskisehir, Trabzon, and Erzurum) participated in the study. The selected cities represent 45.6% of the country′s population according to the 2018 census. Local ethics committee approval was obtained from the Eskisehir Osmangazi University Clinical Research Ethics Committee (2 January 2018; 80558721/48).

Children and adolescents aged between 0 and 18 years were enrolled, and they were planned to be taken in approximately the same number and in equal gender distribution from each age group. We obtained consent from the parents of all children and adolescents. The participants or parents were asked to complete a questionnaire. Age, gender, daycare center or school attendance, smoking status, smoking status of the individuals in the household, antibiotic use during the last one and three months, and an upper respiratory tract infection history in the last month were recorded. The participants were considered vaccinated if they had received any meningococcal vaccines.

Nasopharyngeal samples were taken using cotton swabs (Copan Diagnostics, Carlsbad, California, United States) and placed in AMIES medium with activated charcoal (DeltaLab, Barcelona, Spain). These were transferred to the laboratory with charcoal amies transport tubes. DNA isolation, evaluation of the presence of *Nm*, and serogroup definition in all *Nm*-positive samples were performed.

### 2.2. Laboratory Analysis

Phosphate buffer saline (PBS) solution (1000 μL) was added to the microtube first. Mixing was performed by placing the swabs in PBS solution. After waiting for about 1–2 min, the swab was removed from the solution. DNA isolation was performed using the QuickGene DNA Whole Blood Kit (DB-S, Osaka, Japan) with the QuickGene-Mini80 semi-automatic device. For DNA isolation from PBS fluid, 200 μL of the sample was treated with 250 µL of LDT (lysis buffer) and 30 µL of EDT (Proteinase K). Vortex and short-term spins were performed for 15 s. After 5 min of incubation at 56 °C, short-term spins were made. Then, 250 µL of 99% cold ethanol was added and vortexed for 15 s. After the lysate was thoroughly mixed, all content was transferred to QuickGene cartridges. The binding, washing, and elution steps were performed using the QuickGene device. DNA was reconstituted with 100 µL elution buffer. DNA samples obtained as a result of the study were stored at −80 °C for long-term storage. Single-tube, multiplex PCR analysis was performed for the simultaneous detection of bacterial agents. In each analysis, the final reaction mixture was 22 μL, which was adjusted to have 10 µL of DNA. For the 1X PCR reaction, 1 μL of 2.5 pmol primer and DiagenT11.1 (Diagen Biotech., Ankara, Turkey) buffer mix was prepared as 11 μL. PCR analysis was performed using the Applied Biosystems Veriti 96 Well Thermal Cycler (USA) under the following conditions: 1 cycle at 95 °C for 5 min after the first denaturation, 40 cycles at 95 °C for 1 s, 61 s at 61 °C, 5 s at 72 °C, and 1 cycle as the final elongation at 72 °C for 5 min. General screening was performed with CtrA and PorA. In this study, the sample found positive for *Nm* was reserved for serogrouping [A (Orf-2), B (Sia D), C (Sia D), Y (Sia D), X (CtrA), and W (Sia D)]. We used 10 µL of DNA in the final volume of 22 µL in the detection of serogroups. The 1X PCR reaction was prepared as 1 µL from the 2.5 pmol primer and the DiagenT11.1 (Diagen Biotech., Ankara, Turkey) buffer Mix 11 µL. All products obtained [A (Orf-2), B (Sia D), C (Sia D), Y (Sia D), X (CtrA), and W (Sia D)] were analyzed using 2% agarose gel. Positive and negative controls were used for verification throughout the entire study. In the serogrouping study, verification was performed using the *Nm* real-time PCR serogrouping kit to confirm the weak bands.

### 2.3. Statistical Analysis

The sample size calculated with a power 90% and an alpha error 5% was 1310 people with an estimated prevalence of meningococcal carriage of 10%. Frequency analysis was performed and a 95% confidence interval (CI) for the proportions was calculated. Chi-square test and Mann–Whitney U tests were used for comparisons between groups. All statistical analyses in the study were conducted using SPSS for Windows 11.5 (Chicago, IL, USA). *p* < 0.05 was considered statistically significant.

## 3. Results

In this study, we enrolled 1267 (643 girls and 624 boys) children and adolescents aged between 0 and 18 years old from 13 centers in 12 different cities between January and June 2018. When the subjects participating in the study were divided into age groups, 363 (28.6%) were in the 0–5 age group, 379 (30%) were in the 6–10 age group, 278 (21.9%) were in the 11–14 age group, and 247 (19.5%) were in the 15–18 age group. A total of 897 (70.9%) of the cases participating in the study went to daycare centers or schools. A total of 746 (58.8%) patients had a history of cigarette exposure in the place in which they live, 675 cases (53.3%) had a history of upper respiratory tract infection in the past 1 month, and 533 cases (42.1%) had a history of upper respiratory tract infection in the past 1 week. Antibiotic use history in the last month was reported by 12.4% participants (*n* = 157) and 41.6% (*n* = 527) reported a history of antibiotic in the last 3 months.

*Nm* carriage was detected in 96 (7.5%, 95% CI 6.1–9.0) of the participants. The mean age of the patients with *Nm* carriage was 9.8 ± 5.1 years, and the mean age of the cases without carriage was 9.1 ± 4.9 years (*p* > 0.05), with the peak age at 13 years (12.5%). *Nm* carriage was found to be higher in the 11–14 and 15–18 age groups than in the 0–5 and 5–10 age groups, but the difference was not statistically significant (*p* > 0.05). Of the 96 cases with meningococcal carriage, 49 were female (51%) and 47 were male (49%). No significant difference was found between the two groups with and without *Nm* carriage (*p* > 0.05).

**Figure 1:**
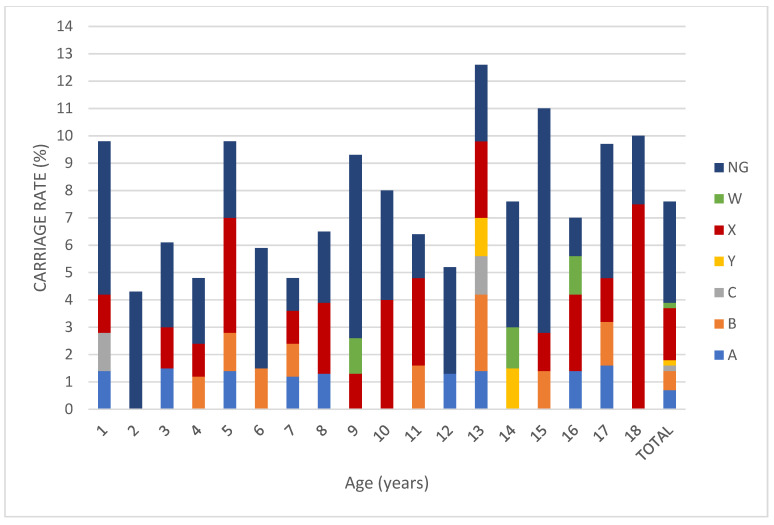
Percentage of meningococcal serogroup carriage among children and adolescents aged between 0 and 18 years old. (NG: non-groupable).

**Figure 2:**
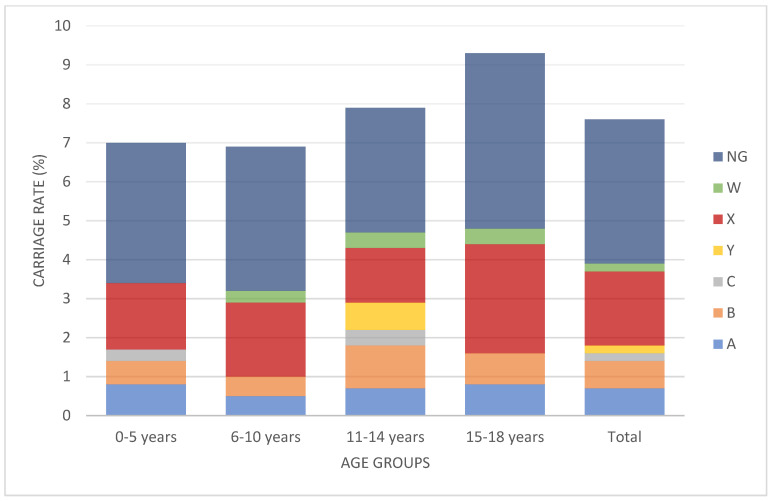
Percentage of meningococcal serogroup carriage (including non-groupable isolates) according to age group. There were no statistical differences between the age groups (*p* > 0.05). (NG: non-groupable).

The serogroup distribution of the children in whom *Nm* carriage was detected was as follows: nine (9.4%) for MenA, nine (9.4%) for MenB, two (2.1%) for MenC, three (3.1%) for MenW, 24 (25%) for MenX, two (2.1%) for MenY, and 47 (48.9%) for non-groupable meningococci. When we evaluated groupable serogroups (excluding non-groupable isolates) (*n* = 49), 48.9% of the serogroup was MenX (Figure 1). Regarding age groups, Nm carriage rate was 7% in the 0–5 age group, was 6.9%in the 6–10 age group, was 7.9% in the 11–14 age group, and was 9.3% in the 15–18 age group. There is no statistically significant difference between the groups (*p* > 0.05) (Figure 2).

Forty-three (3.4%) of the cases participating in the study were vaccinated with a meningococcal vaccine; all of these children received a quadrivalent meningococcal conjugate vaccine (MenACWY conjugated vaccines), whereas 1223 did not receive a meningococcal vaccine. Meningococcal carriage was detected in two (4.7%) of the children with a history of vaccination and 94 (7.7%) of the children without a history of vaccination. While there was a low number of vaccinated cases, there was no statistically significant difference in terms of *Nm* carriage between groups that received and did not receive a meningococcal vaccine (*p* > 0.05).

The average number of household members of meningococcal carriers was found to be 4.92 ± 1.9, whereas that of non-meningococcal carriers was 4.2 ± 1.5 (*p* < 0.05). There was no statistically significant difference between groups with and without meningococcal carriage in terms of exposure to cigarette smoke (*p* > 0.05). Meningococcal carriage was detected in 5 (3.2%) of 157 patients with a history of antibiotic use in the last month and in 91 (8.2%) of 1109 patients without a history of antibiotic use in the last month. A statistically lower carriage was found in the group using antibiotics compared with the group not using it (*p* < 0.05). Meningococcal carriage was detected in 30 of 527 patients (5.7%) who had a history of antibiotic use in the last three months and in 66 (8.9%) of 738 patients without a history of antibiotic use in the last three months (*p* > 0.05). Of the 675 patients who had upper respiratory tract infection in the last month, 62 (9.1%) had meningococcal carriage. Of the 591 cases with no history of upper respiratory tract infection in the last month, 36 (6%) had meningococcal carriage. The carrier rate was found to be higher in those patients with a history of infection (*p* < 0.05). When the cases were evaluated in terms of school or daycare center attendance, it was found that 897 cases continued going to the school or daycare center, and 63 (7%) of these had meningococcal carriage. There were 369 cases without school or nursery attendance, and 33 (8.9%) of these had meningococcal carriage. When the cases were compared in terms of school or nursery school, no statistically significant difference was found between those attending school or nursery school and those who did not attend nursery school (*p* > 0.05).

## 4. Discussion

In this study, the overall meningococcal carriage rate was 7.5% among children who are 0–18 years old, with the peak age at 13 years (12.5%), and 9.3% among children who are 5–18 years old. Serogroup X was the predominant serogroup, followed by MenB and MenA, whereas 49% was non-groupable/unencapsulated. *Nm* carriage rate was associated with previous-month upper respiratory tract infection, a high number of household members, and antibiotic use in the last month.

**Figure 3:**
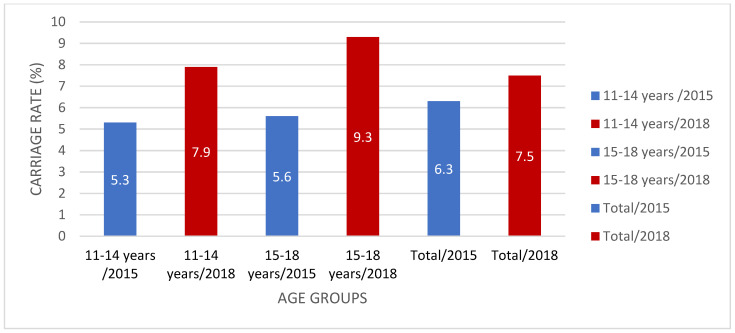
Meningococcal carriage rate (%) comparison in Turkey between 2015 and 2018.

The meningococcal carriage rate in the present study was higher (7.5%) than that in our 2015 study (6.3%) in the same cities in Turkey [15]. Our previous study [15] included children, adolescents, and young adults aged between 10 and 24 years old, whereas the current study included 0–18-year-olds. When we compare the matched age groups, in 2015, the carriage rate was 5.3% in the 10–14 age group and 5.6% in the 15–18 age group, whereas it was 7.9% and 9.3% in the present study, respectively [15] (Figure 3). In light of these data, it can be said that there has been an increase in *Nm* carriage during adolescence in Turkey in the past few years, and *Nm* carriage has been observed in all age groups from 0 to 18 years old.

The rate of meningococcal carriage was 7.5% in our study, with no significant differences detected between early childhood and adolescence. Meningococcal carriage increased with age, with low carriage in young children and increasing to 23.7% in 19-year-olds [7]. Carriage rates vary over the world, with 7.3% in Norway (12–24 years old) [8], 4% in Greece (2–19 years old) [16], 9% in Brazil (1–24 years old) [17], and 16% in the Netherlands (13–23 years) [18]. Recently, the rate of *Nm* carriage in Paraguay was low (2.1%) among individuals aged 3 to 21 years [19]. In the Americas, the carriage rate ranged from 1.6% to 9.9%, whereas in Asia, it ranged from 1.4% to 14.2% [9]. In North America and Europe comparing adolescent time, the carriage rates during the first year of life are quite low, presumably because of social behavior throughout adolescence [6]. The highest carriage rates were found in those aged 5–14 years old in a recent longitudinal investigation of *Nm* carriage in Sub-Saharan Africa [20]. While age is a significant component in *Nm* carriage, the rate of *Nm* carriage varies by geographical region and may be influenced by other factors. Living in overcrowded environments (e.g., dormitories), intercurrent viral respiratory tract illness, active or passive smoking or smokeless tobacco, intimate contact, and, frequenting bars or clubs have all been identified as factors that increase the probability of carriage. *Nm* carriage rate was linked to previous-month upper respiratory tract illness, antibiotic use in the preceding month, and the presence of many household members in this study [4,6,7,8,9,10,11,15,21]. Spyromitrou-Xioufi et al. [22] recently published a systematic review and meta-analysis about the risk factors for meningococcal disease in children and adolescents. Household crowding, smoking exposure, close relationships, and recent respiratory tract infections increased the risk of IMD in exposed individuals. In a prior study, individuals who had an upper respiratory tract infection in the previous three months had a statistically significantly increased carrier rate [15]. Cassio de Moraes et al. [10] showed a link between having had an upper respiratory tract infection and meningococcal carriage in the previous two weeks in Brazil. Routine immunization could have an impact on IMD and *Nm* carriage epidemiology, but vaccination with MCV4 (3.4% in our study) had no effect on carriage. The effects of MCV4 on carriage and herd immunity need to be investigated further [23,24].

The serogroup distribution of *Nm* carriage also differs by geographical region. In Latin America, Serogroups C and Y were found to be the most frequent. Serogroups B, Y, and others were the most prevalent in the majority of the European continent. MenW, MenX, MenY, and others (non-ABCWXY and non-groupable) were the most common in Africa according to 21 studies [21]. In our previous investigation in Turkey, Serogroup W was the most commonly carried serogroup [15], and it was also a notable serogroup discovered in a carriage study in England in 2015–2016 [25]. The serogroup distribution in this study revealed that 48.9% of the meningococci were non-groupable, 25% were MenX, 9.4% were MenA, 9.4% were MenB, 3.1% were MenW, 2.1% Men Y, and 2.1% were MenC. Around half of the *Nm* strains were non-groupable in our study, a result that is similar to that in our previous study in 2015 and other recent studies [15,21]. When non-groupable isolates were removed, 48.9% of the serogroup was MenX, which was an unexpected finding. In Turkey, Men X is a sporadically reported cause of IMD. Between the two study periods, no routine pediatric meningococcal vaccine was administered in Turkey. Vaccination was performed in limited numbers, only in private practice, we do not think that this vaccination rate may have an effect on the carrier rates. In 2010, a soldier diagnosed with meningococcal meningitis was the first known MenX case in Turkey [26]. In 2017, the first pediatric case of Serogroup X was discovered in Ankara as part of a meningococcal surveillance study [27]. Worldwide, sporadic cases of IMD caused by MenX have been identified; according to PubMLST databases, 636 MenX isolates were reported between 1961 and 2019 [3]. In the 1990s, MenX was identified as a source of IMD epidemics in Sub-Saharan African nations, such as Kenya, Niger, Ghana, and Burkina Faso. MenX has been more visible in the African Meningitis Belt since the launch of the MenA conjugate vaccine. The disappearance of MenA in Ghana following the introduction of the MenA conjugate vaccine was accompanied by a rapid increase in MenX carriage, which reached 17% and coincided with a MenX disease outbreak. MenX (cc750)-related IMD has also been reported in Europe [3]. The MenX carriage rate in our study was higher than that in earlier trials. Men X isolates have also been reported among teenagers in European countries (e.g., Norway, 2.4%) [8]. Malaysia has a higher Men X carriage rate; Rohani et al. [28] found that 81.0% of the isolates from 3195 army recruits aged 17–24 years who were receiving intensive training in an army camp in 2005 were due to MenX/Z. Differences in vaccination policy, the creation of hypervirulent clones, natural immunity, exposure to different settings, and behavioral risk factors may explain the differences between countries and fluctuations in serogroup distribution. While the Men X carriage rate is higher than expected, there is no increase in the number of IMD patients in Turkey as a result of MenX [13]. According to recent research, MenX has a lower disease-to-carriage ratio than MenA, as well as a reduced invasive potential [3]. The capsular change in meningococci may have resulted in the high Serogroup X carriage in our study. In China, Ji et al. [29] showed that capsule switching happens at a high frequency and occurs in an emerging sequence Type 7 Serogroup X strain. In the literature, however, there is no information on the capsular switch between MenW (a previously circulating viral serogroup in Turkey) and MenX. There are current clinical phase trials using a pentavalent meningococcal conjugate vaccination (for MenA, C, Y, W, and X) for the potential control of MenX disease/outbreak [30,31]. Two formulations, with and without adjuvant, seemed immunogenic and had an acceptable safety profile in a Phase 1 clinical investigation in the US [30]. A serum bactericidal antibody titer of at least 128 was seen in 91%–100% (for all five serotypes) of the patients in a Phase 2 randomized controlled study including Malian children aged 12 to 16 months [31]. Further research into MenX-containing vaccinations could provide a remedy to possible outbreaks.

One limitation of this study is that we did not perform routine culture for the detection of *Nm*, and we had no chance to perform sequence analysis. When PCR is used for detection and serogrouping, carefully selecting the primers to avoid inadvertently underestimating carriage is important. The addition of primers for other targets, such as SodC and/or PorA, might be appropriate [21]. In this study, screening was conducted with CtrA and PorA.

## 5. Conclusions

Meningococcal carriage studies have been conducted to determine the prevalence of carriage and serogroup distribution and thus better understand transmission dynamics and IMD epidemiology and improve vaccination strategies. Compared with the 2015 study results, those of the present research show that Serogroup X carriage rate was higher than expected, and the Serogroup W carriage rate was low [15]. There is no Serogroup X predominance for IMD in Turkey. For this reason, at this stage there is no indication for potential use of MenX-containing vaccines. The geographical distribution of *Nm* strains varies, but the serogroup distribution in the same country can shift over time. For accurate/dynamic serogroup distribution and prospective vaccine options, adequate surveillance and/or thorough carriage studies are essential. We planned to conduct the third part of the carriage study in 2020, but because of the pandemic, we had to postpone it until the end of 2021. For a probable serogroup change, capsular switching, and outbreak situation, vaccination plans against all clinically relevant *Nm* should be developed.

## Data Availability

Not available.
